# Ahnak promotes tumor metastasis through transforming growth factor-β-mediated epithelial-mesenchymal transition

**DOI:** 10.1038/s41598-018-32796-2

**Published:** 2018-09-26

**Authors:** Mira Sohn, Sunmee Shin, Jung-Yeon Yoo, Yookyung Goh, In Hye Lee, Yun Soo Bae

**Affiliations:** Department of Life Sciences, Ewha Womans University, Seoul, 120-750 Korea

## Abstract

Previously, we reported a molecular mechanism by which Ahnak potentiates transforming growth factor-β (TGFβ) signaling during cell growth. Here, we show that Ahnak induces epithelial-mesenchymal transition (EMT) in response to TGFβ. EMT phenotypes, including altered in cell morphology, and expression patterns of various EMT marker genes were detected in HaCaT keratinocytes transfected with Ahnak-specific siRNA. Knockdown of Ahnak expression in HaCaT keratinocytes resulted in attenuated cell migration and invasion. We found that Ahnak activates TGFβ signaling via Smad3 phosphorylation, leading to enhanced Smad3 transcriptional activity. To validate function of Ahnak in EMT of B16F10 cells having high metastatic and tumorigenic properties, we established B16F10 cells with stable knockdown of Ahnak. N-cadherin expression and Smad3 phosphorylation were significantly decreased in B16F10-shAhnak cells, compared to B16F10-shControl cells after treatment of TGFβ. Moreover, TGFβ failed to induce cell migration and cell invasion in B16F10-shAhnak cells. To determine whether Ahnak regulates the metastatic activity of B16F10 cells, we established a lung metastasis model in C57BL/6 mice via tail vein injection of B16F10-shAhnak cells. Lung metastasis was significantly suppressed in mice injected with B16F10-shAhnak cells, compared to those injected with B16F10-shControl cells. Taken together, we propose that TGFβ-Ahnak signaling axis regulates EMT during tumor metastasis.

## Introduction

Tumor metastasis comprises multiple steps, including the generation of circulating tumor cells (CTCs) from the primary tumor, the dissemination of CTCs into target tissue to generate a secondary tumor, and metastatic colonization^1–3^. The CTC dissemination process can be subdivided into intravasation, transport through circulation, arrest at a distant secondary tissue, and extravasation^1–3^. Because many complex proteins are involved in tumor metastasis, the detailed molecular mechanism of metastasis is still unclear. Epithelial-mesenchymal transition (EMT) seems to be one complex molecular process involved in the initial development of tumor metastasis^4–6^. Loss of epithelial properties, including apical-basal polarity and cell-cell adhesion, in the primary tumor leads to gain of mesenchymal cellular function, with increased cell migration and invasive activity.

Various cytokines, including transforming growth factor β (TGFβ), are known to regulate the EMT process in metastasis^7–9^. TGFβ acts as a multifunctional cytokine in cell growth and in the regulation of EMT during tumor metastasis. TGFβ binds to heterodimeric type II and type I receptors, and the TGFβ type II receptor then phosphorylates and activates the TGFβ type I receptor. The activated type I receptor phosphorylates receptor-regulated Smads (R-Smads), leading to an association with a common partner Smad (co-Smad). The heterodimeric complex of R-Smad and co-Smad translocates into the nucleus and regulates the transcription of target genes. It has been well established that TGFβ regulates EMT during tumor metastasis by controlling the expression of Smad3-mediated target genes. In particularly, the TGFβ/Smad3 signaling cascade regulates the expression of the Snail/Slug, ZEB1/2 and Twist families during EMT and the secretion of metalloproteases (MMPs), endowing invasive properties to mesenchymal cells^10^.

Ahnak has been reported as a mysterious, giant scaffolding protein^11^. Previously, we reported that Ahnak binds and activates phospholipase C-γ1 and PKC in response to stimulation with a growth factor such as PDGF or EGF^12–15^, resulting in the regulation of smooth muscle cell migration. Thus, Ahnak appears to be a molecular link between inositide-mediated calcium mobilization and growth factor stimulation. Moreover, we have recently determined that Ahnak acts as a tumor suppressor by activating the TGFβ/Smad3 signaling cascade, which leads to cell cycle arrest in G0/G1 phase and downregulation of c-Myc expression during cell growth^16^. These results indicated that Ahnak serves as a molecular link between inositide-mediated cell signaling and cell growth and migration and between cytostatic activity and the regulation of TGFβ/Smad3 signaling.

Although TGFβ is known to be a cytostatic effector in pre-malignant cells, it also serves as an enhancer of the invasion and metastasis of advanced carcinoma cells^7–10^. Several lines of evidence have indicated that Ahnak is involved in cell migration and metastasis^17–19^. Many proteomics datasets have suggested that Ahnak expression is enhanced in various metastatic cancer tissues. Interestingly, TGFβ-mediated EMT of A549 lung cancer cells stimulates Ahnak expression^20^. A recent report showed that Ahnak is involved in the EMT process and is required for pseudopod protrusion in various cancer cell lines^18^. Moreover, Ahnak expression is highly associated with the metastasis of an aggressive mesothelioma tumor^17^. However, the molecular mechanism by which Ahnak is involved in tumor metastasis is unclear. Here, we report the molecular mechanism of Ahnak function in EMT and extravasation through activation of TGFβ/Smad3 signaling cascade.

## Results

### Ahnak regulates TGFβ-induced EMT

**Figure 1:**
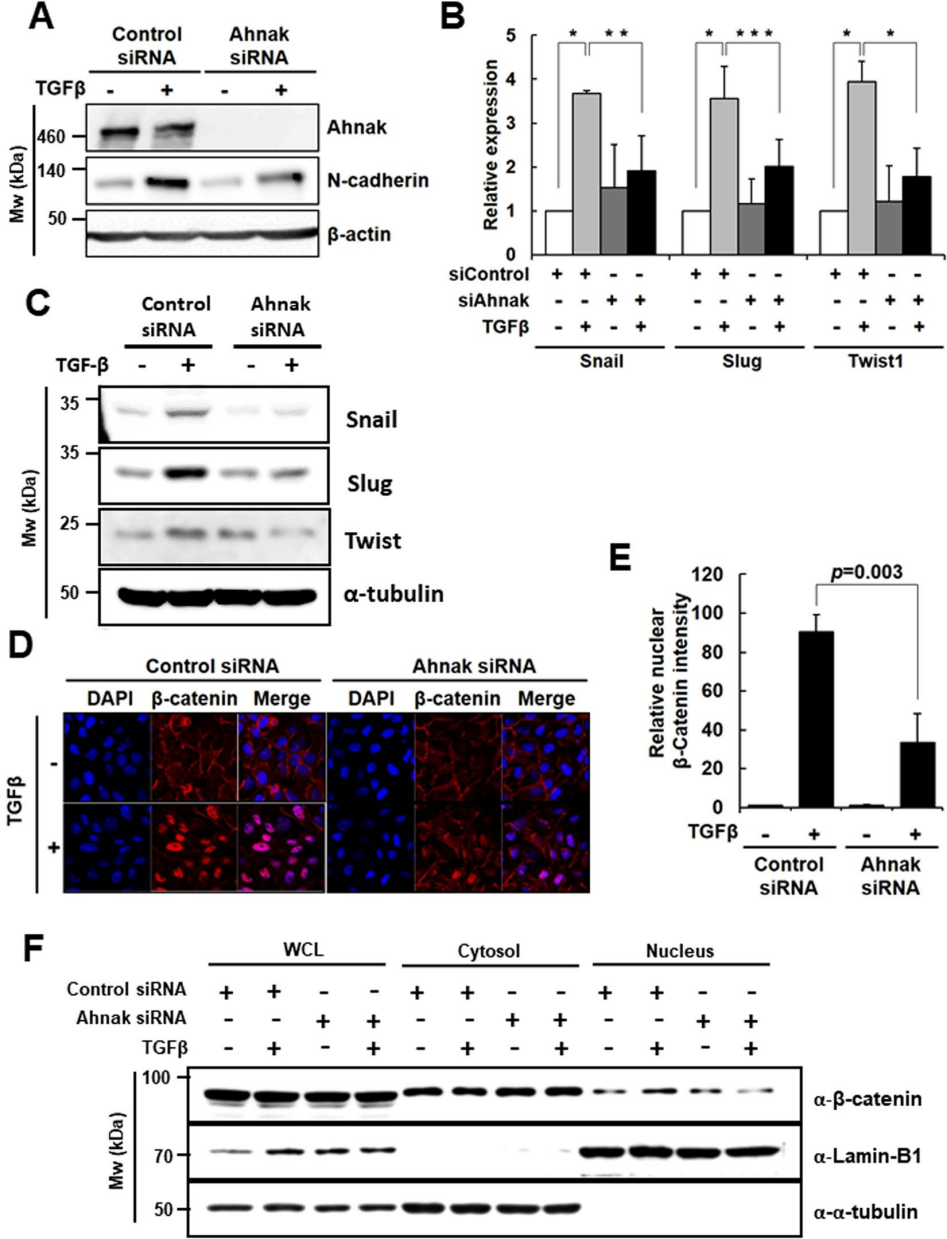
Requirement of Ahnak for TGFβ-induced EMT. (**A**) HaCaT cells were transfected, serum deprived, and stimulated with TGFβ (5 ng/mL) for 72 h. Cells were lysed and subjected to western blot analysis with the indicated antibodies. (**B**) HaCaT cells were transfected with the indicated siRNA, serum-deprived, and stimulated with TGFβ (5 ng/mL) for 48 h. Total RNA was isolated, and the expression of each gene was quantified with qRT-PCR. The average fold increase, S.D., and statistical significance (*P*-value from Student’s t-test) were obtained from three independent experiments (*p < 0.01, **p < 0.02, ***p = 0.05). (**C**) HaCaT cells were transfected with the indicated siRNA, serum-deprived, and stimulated with TGFβ (5 ng/mL) for 24 h. Cells were lysed and subjected to western blot (WB) analysis with the indicated antibodies. (**D**) HaCaT cells were transfected, serum deprived and stimulated with TGFβ (5 ng/mL) for 48 h. Cells were stained with an antibody against β-catenin (red) and with DAPI (blue). Fluorescence images were captured with a confocal microscope (600X). (**E**) Nuclear β-catenin was quantified by comparing the red fluorescence intensity per cell. (**F**) HaCaT cells (6 × 10^4^) cultured in 100 mm culture dishes were transfected control siRNA or Ahnak siRNA as described above. HaCaT cells were serum starved for 16 h and then incubated with or without TGF-β (5 ng/ml) for 1 h. Lamin-B and tubulin serve as nuclear and cytosol marker protein. The nuclear fraction was isolated by the cell fractionation kit (Cell signaling; #9038) according to manufacturer’s instruction.

Previously, we reported that Ahnak stimulates TGFβ-induced cell signaling through R-Smad activation, leading to suppressed cell growth^16^. Interestingly, TGFβ is known to be involved in the EMT. Therefore, we tested whether the Ahnak-TGFβ axis mediates EMT. HaCaT cells were transfected with siRNA specific for Ahnak (Ahnak siRNA/HaCaT), and the Ahnak knockdown efficiency was measured by immunoblot assay (Fig. 1A and Supplementary Fig. S1). To verify the function of Ahnak in the TGFβ-induced EMT of HaCaT cells, we analyzed the expression of N-cadherin, a mesenchymal cell marker, after treatment with TGFβ. N-cadherin expression was up-regulated in control siRNA-transfected HaCaT (control siRNA/HaCaT) cells stimulated with TGFβ but was unchanged in Ahnak siRNA/HaCaT cells under the same conditions (Fig. 1A). Moreover, knockdown of Ahnak expression in HaCaT cells significantly reduced the expression of three EMT master transcription factors (Snail, Slug (Snail2), and Twist1) in response to TGFβ compared to that in control cells (mRNA level for Fig. 1B, protein level for Fig. 1C and Supplementary Fig. S2). Because β-catenin translocation from the plasma membrane into the nucleus is another EMT phenotypic event, we assessed the nuclear localization of β-catenin in Ahnak siRNA/HaCaT cells after stimulation with TGFβ and found it to be attenuated compared to that in control siRNA/HaCaT cells (Fig. 1D,E). Moreover, we performed fractionation of cytosol and nucleus and measured β-catenin level in cytosol and nucleus of Ahnak siRNA/HaCaT and control siRNA/HaCaT cells in the presence and absence of TGFβ. We found nuclear translocation of β-catenin protein was attenuated in Ahnak siRNA/HaCaT, compared to that in control siRNA/HaCaT cells (Fig. 1F and Supplementary Fig. S3). These results indicate that Ahnak is required for EMT driven by TGFβ.

### Ahnak regulates cell migration and invasion in response to TGFβ

**Figure 2:**
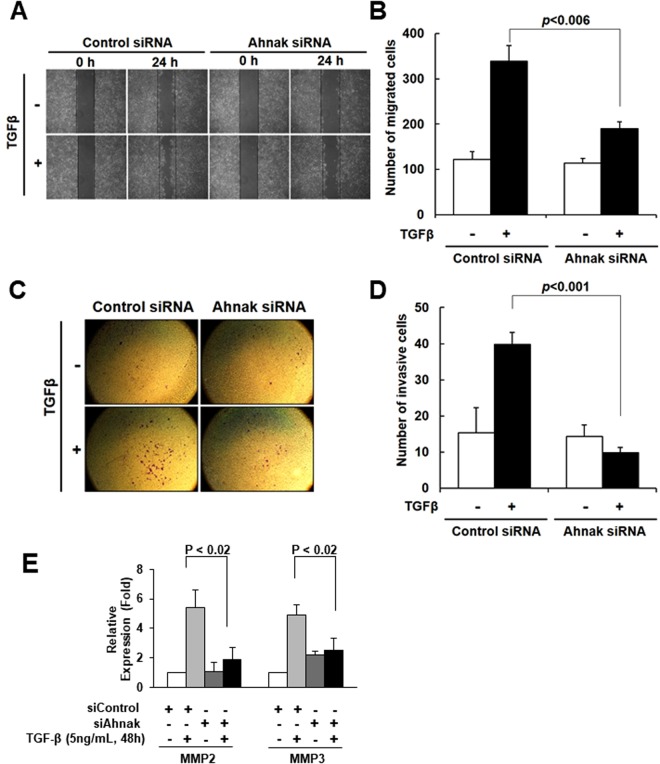
Essential role of Ahnak in EMT-mediated cell migration and invasion. (**A**,**B**) HaCaT cells were transfected with the indicated siRNA, serum-starved, and treated with mitomycin C (10 μg/mL), a proliferation inhibitor, for 3 h. Cells were subjected to wound healing assays with or without 5 ng/mL of TGFβ for 24 h and the number of migrated cells (B) was counted. (**C**,**D**) HaCaT cells were transfected with the indicated siRNA, serum deprived, and treated with mitomycin C (10 μg/mL) for 3 h. Cells were trypsinized and subjected to Matrigel invasion assays for 48 h in the presence or absence of TGFβ (5 ng/mL). Cells were stained with crystal violet. Invaded cells were observed with an optical microscope (50X). The cell number (**D**) was counted and graphed as the mean ± SD. of three independent Matrigel invasion assays. Statistical significance was calculated using Student’s t-test. (E) HaCaT cells were transfected with the indicated siRNA, serum deprived, and stimulated with TGFβ (5 ng/mL) for 48 h. Total RNA was isolated, and MMP2 and MMP3 genes expression was quantified by qRT-PCR. The average fold increase, S.D. and statistical significance (*P*-value from Student’s t-test) were obtained from three independent experiments.

Because EMT is characterized by cell migration and invasion, we investigated the function of Ahnak in wound healing and matrigel invasion by using Ahnak siRNA/HaCaT cells. In control siRNA/HaCaT cells, cell migration was significantly increased by TGFβ stimulation (Fig. 2A,B). However, Ahnak siRNA/HaCaT cells showed reduced cell migration in response to TGFβ (Fig. 2A,B). The results suggested that Ahnak is essential for TGFβ-induced cell migration. To assess the function of Ahnak in cell invasion, control siRNA/HaCaT and Ahnak siRNA/HaCaT cells were treated with mitomycin C (10 μg/mL), and the cells were then plated onto matrigel matrix. TGFβ stimulation of control siRNA/HaCaT cells resulted in significantly increased cell invasion, whereas Ahnak siRNA/HaCaT cells showed no invasive activity (Fig. 2,D). To identify the molecular drivers of the invasive activity of mesenchymal cells, we measured the expression of MMP2/3, which is involved in the dissemination of CTC, in cells treated with TGFβ. Stimulation of Ahnak siRNA/HaCaT cells with TGFβ resulted in a significant decrease in MMP2/3 expression compared to that in control siRNA/HaCaT cells (Fig. 2E). Taken together, our data show that Ahnak regulates TGFβ-induced cell migration and invasion.

### Ahnak-mediated TGFβ signaing in EMT

**Figure 3:**
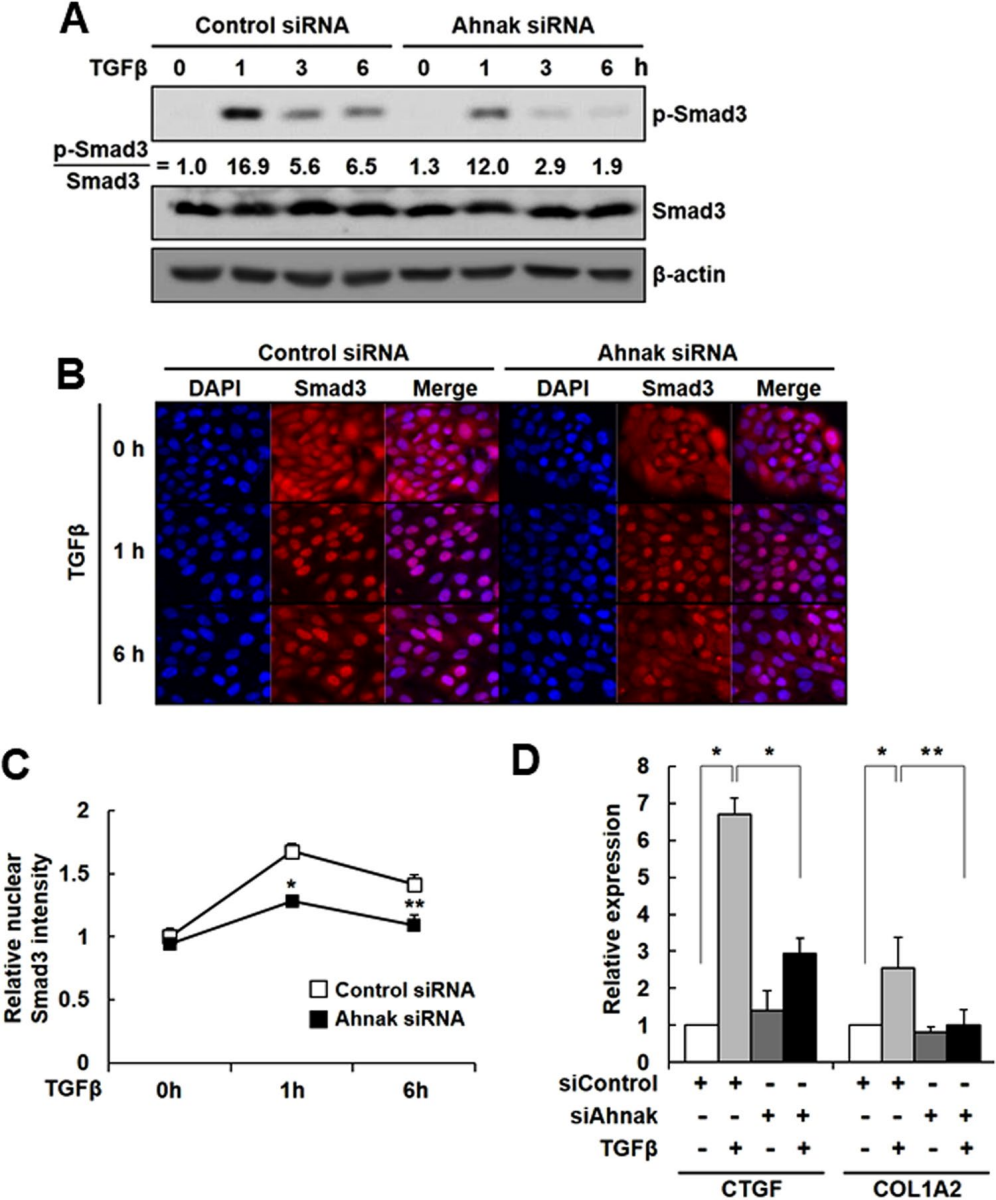
Activation of TGFβ/Smad3 signaling by Ahnak. (**A**) HaCaT cells were transfected with the indicated siRNA, serum starved, and stimulated with TGFβ (5 ng/mL) for the indicated time. Cells were lysed and subjected to western blot analysis with antibodies to p-Smad3, Smad3, and actin. (**B**) HaCaT cells were transfected with the indicated siRNA, serum starved, and stimulated with TGFβ (5 ng/mL) for 0, 1, and 6 h. Cells were stained with anti-Smad3 (red) antibody and DAPI (blue). Fluorescent images were obtained using a confocal microscope (600X). (**C**) Nuclear Smad3 was quantified by comparing red fluorescence intensity (nuclear Smad3/all intracellular Smad3) (*p < 0.001, **p < 0.03). (**D**) Smad3 target gene expression. HaCaT cells were transfected with the indicated siRNA, serum deprived, and stimulated with TGFβ (5 ng/mL) for 48 h. The gene expression of direct Smad3 targets was quantified by qRT-PCR. The mean ± S.D. and statistical (Student’s t-test) were determined based on three independent experiments (*p < 0.01, **p < 0.03).

We previously identified Ahnak as a positive regulator of the TGFβ-Smad3 signaling cascade, which leads to the suppression of cell growth^16^. We evaluated whether Ahnak positively regulates TGFβ-induced EMT in a Smad3-dependent manner. Knockdown of Ahnak expression in HaCaT cells significantly decreased Smad3 phosphorylation in response to TGFβ compared to control knockdown (Fig. 3A and Supplementary Fig. S4). We explored whether Ahnak regulates the TGFβ-mediated nuclear translocation of Smad3. Stimulation of control siRNA/HaCaT cells with TGFβ resulted in increased nuclear translocation of Smad3, whereas this translocation event was attenuated in Ahnak siRNA/HaCaT cells (Fig. 3B,C).

Next, we questioned whether Smad3 signaling via the TGFβ-Ahnak signaling axis stimulates Smad3-dependent target gene expression related to EMT. Connective tissue growth factor (CTGF) and collagen 1A2 (COL1A2) have been reported as Smad3-mediated target genes during TGFβ-induced EMT^4–6^. TGFβ stimulation of Ahnak siRNA/HaCaT cells significantly decreased the expression of two genes (CTGF and COL1A2) compared to stimulation of control siRNA/HaCaT cells (Fig. 3D). These results suggest that Ahnak regulates the TGFβ-induced activation of Smad3 and the expression of Smad3 target genes in EMT.

### Effect of Ahnak on TGFβ-mediated EMT in B16F10 melanoma cells

**Figure 4:**
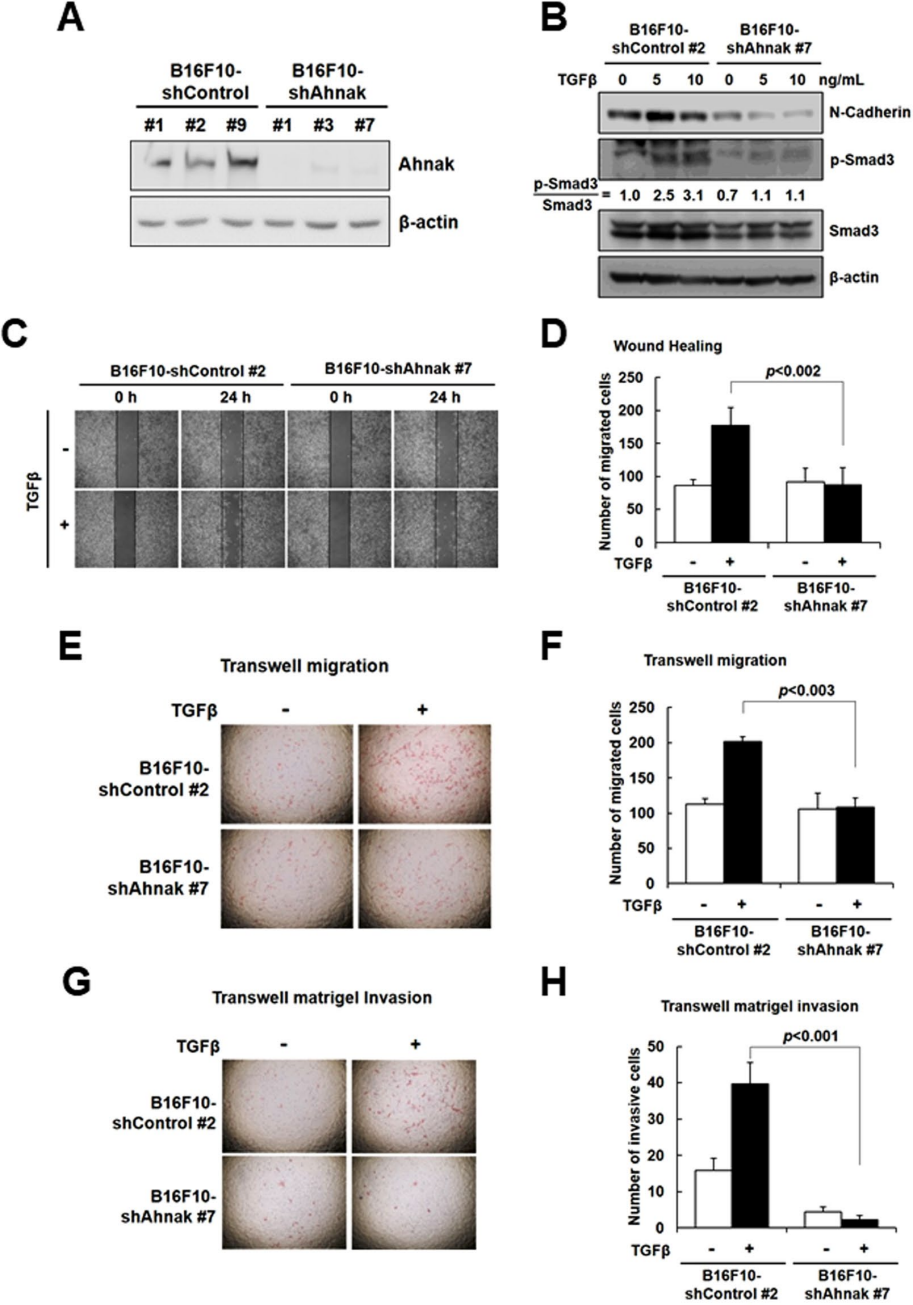
Ahnak regulates EMT-mediated melanoma cell migration and invasion. (**A**) B16F10 melanoma cells were stably infected with pGIPZ lentivirus-shControl and shAhnak and selected by colony picking to generate monoclonal cell line. Each cell line was subjected to western blot analysis with the indicated antibodies. (**B**) Ahnak-depleted B16F10 stable cells were serum starved and stimulated with TGFβ (5 ng/mL) for 24 h. Cells were lysed and subjected to western blot analysis with the indicated antibodies. (**C**) Ahnak-depleted B16F10 cells were serum starved and treated with mitomycin C (10 μg/mL), a proliferation inhibitor, for 3 h. Cells were subjected to wound healing assays with or without 5 ng/mL TGFβ for 24 h, and the number of migrated cells was counted. (**D**) The number of migrated cells was from (C). (**E**) Ahnak-depleted B16F10 cells were serum deprived, and treated with mitomycin C (10 μg/mL) for 3 h. The cells were subjected to Transwell migration assays for 24 h. (**F**) Graph shows quantification of number of migrated cells from (**E**). (**G**) Matrigel invasion assays for 48 h in the presence or absence of TGFβ (5 ng/mL). Cells were stained with H&E for observation, and the number of migrated cells is presented graphically as mean ± S.D. (**H**) Graph shows quantification of number of invaded cells from (**G**).

Because HaCaT cells are non-tumorigenic and non-metastatic, we investigated the effect of Ahnak on TGFβ-mediated EMT process in B16F10 melanoma cells, which have high metastatic and tumorigenic properties. To validate the function of Ahnak in EMT in B16F10 cells, we established B16F10 cells with stable knockdown of Ahnak. B16F10 cells were infected with pGIPZ lentivirus harboring shControl or shAhnak, and stable cell lines were selected (control cell lines: B16F10-shControl #1, #2, and #9; Ahnak cell lines: B16F10-shAhnak #1, #3, and #7). Ahnak expression in B16F10 cells was measured by western blot analysis (Fig. 4A and Supplementary Fig. S5A). Among the generated cell lines, we selected B16F10-shControl #2 and B16F10-shAhnak #7 cells, both of which were assayed for N-cadherin expression, cell migration, and cell invasion in response to TGFβ. N-cadherin expression and Smad3 phosphorylation were significantly decreased in B16F10-shAhnak #7 cells compared to B16F10-shControl #2 cells after treatment of TGFβ (Fig. 4B and Supplementary Fig. S5B).

To ascertain the effect of Ahnak on cell migration, B16F10-shControl #2 and B16F10-shAhnak #7 cells were subjected to wound healing assay (Fig. 4C,D) and transwell migration assays (Fig. 4E,F). TGFβ suppressed cell migration in B16F10-shAhnak #7 cells compared to B16F10-shControl #2 cells stimulated with TGFβ for 24 h (Fig. 4C–F). To evaluate the effect of Ahnak on cell invasion, mitomycin-treated B16F10-shControl #2 cells and B16F10-shAhnak #7 cells were plated on matrigel. B16F10-shAhnak #7 cells failed to invade the matrigel in response to TGFβ stimulation for 48 h, whereas B16F10-shControl #2 cells showed the expected invasive behavior (Fig. 4G,H). We next measured proliferation rates of B16F10 shAhnak #7 and shControl #2 which remained unchanged for 48 h (Supplementary Fig. S6).

**Figure 5:**
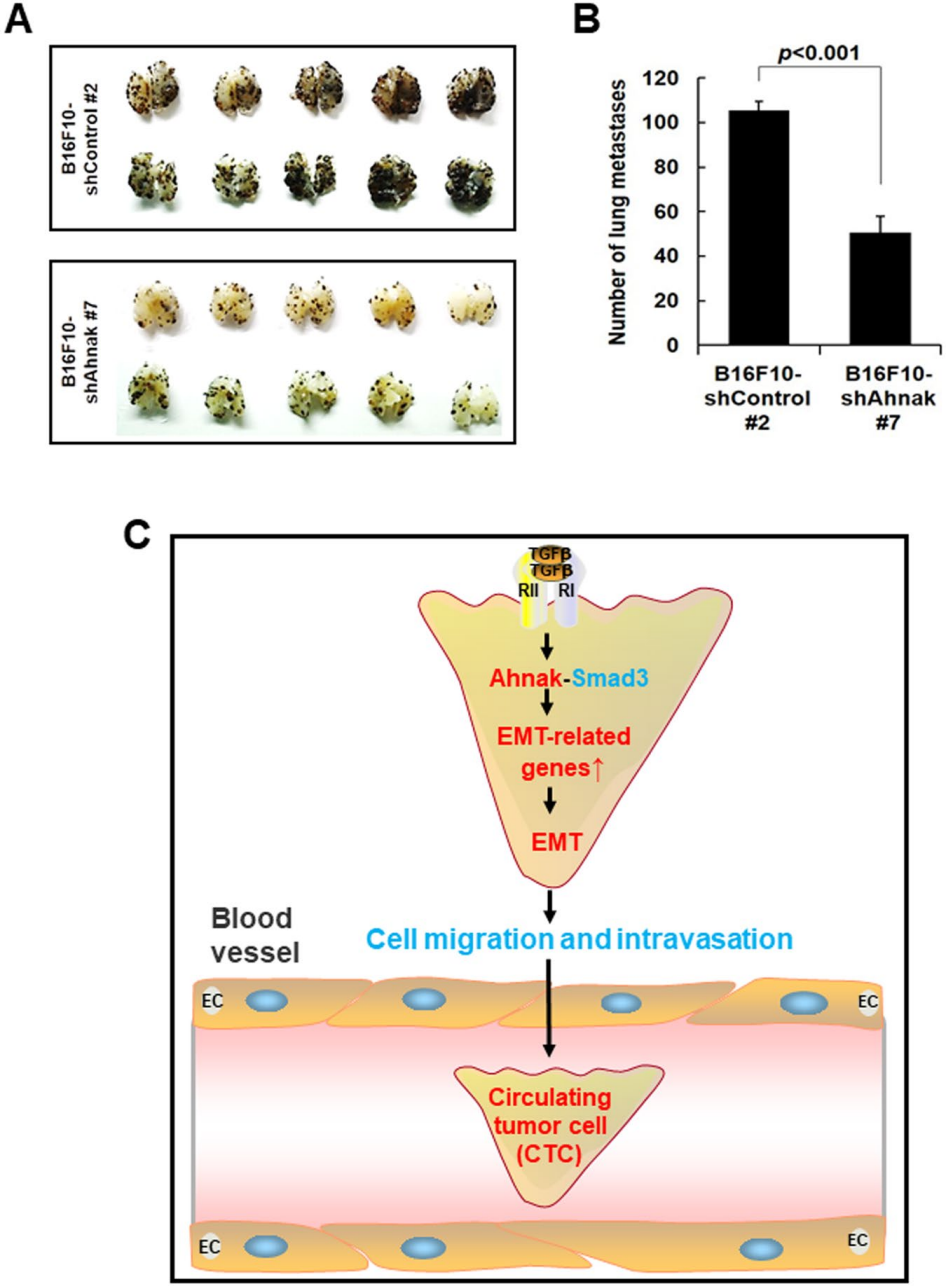
Host function of Ahnak in pulmonary endothelial cells during extravasation of B16F10 melanoma cells. (**A**) Ahnak-depleted stable cells were inoculated into 6- to 8-wk-old male C57BL/6 mice via tail vein injection. The mice were sacrificed fourteen days later. Photographs show the dorsal and ventral sides of lungs containing metastatic lesions. (**B**) The number of metastatic colonies was from (**A**). (**C**) The TGFβ-Ahnak-Smad3 axis stimulates the expression of EMT-related genes and confers cells with motility and invasiveness resulting in intravasation. Taken together, the data indicate that Ahnak plays an important role in cancer metastasis through the regulation of EMT.

To determine whether Ahnak regulates the metastatic activity of B16F10-shAhnak #7 or B16F10-shControl #2 cells *in vivo*, we established a lung metastasis model in C57BL/6 mice via tail vein injection. Mice injected with B16F10-shControl #2 cells had multiple lung metastatic nodules at 14 days (Fig. 5A,B). In contrast, lung metastasis was significantly suppressed in mice injected with B16F10-shAhnak #7 cells (Fig. 5A,B). These results indicate that Ahnak expression is required in B16F10 melanoma cells for pulmonary metastasis.

## Discussion

TGFβ is a well-known cytokine that mediates tumor metastasis through the activation of EMT^21–23^. Cellular and molecular changes during TGFβ-mediated EMT require regulation at the transcriptional, translational, and post-translational levels. The complex EMT process involves the regulation of effector molecule expression, such as the down-regulation of E-cadherin, an epithelial cell marker and the up-regulation of N-cadherin, a marker of mesenchymal cells. EMT also requires the expression of transcriptional factors that coordinate this process, such as Snail/Slug, Zeb1/2, and Twist.

We wondered whether Ahnak regulates TGFβ-induced EMT. Since we have reported that Ahnak is a novel regulator of the TGFβ signaling cascade through its interaction with R-Smad, we hypothesized that Ahnak may induce EMT via the TGFβ-Smad3 signaling cascade. Moreover, many reports have suggested that Ahnak is involved in tumor metastasis^17–20,24^. Therefore, previous reports published by our laboratory and others led us to validate the function of the scaffolding protein Ahnak in tumor metastasis through potentiation of the TGFβ signaling network involved in EMT^17–20,24^. TGFβ-mediated morphological changes and changes in EMT markers were not detected in Ahnak-deficient cells (Fig. 1). Consistently, a previous report showed that Ahnak is essential for mesenchymal characteristics such as pseudopodial protrusion in several metastatic cancer cell lines^18^. Moreover, cell migration and invasion were attenuated in Ahnak-depleted HaCaT cells (Figs 2,3). We previously reported that Ahnak promotes PDGF-induced aortic smooth muscle cell (ASMC) migration due to the concerted action of Erk and Rac^15^. Additionally, we observed that Ahnak is required for the induction of MMP2 and MMP3 in response to TGFβ (Fig. 2E). Therefore, Ahnak seems to modulate the migration and invasion machinery under various physiological conditions. As a novel regulator of TGFβ/Smad3 signaling, Ahnak plays an important role in the expression of Smad3-mediated target genes such as CTGF and COL1A2 (Fig. 3D). Thus, we conclude that Ahnak plays an important role in TGFβ-induced EMT during intravasation (Fig. 5C).

In view of the “seed-and-soil” hypothesis of tumor metastasis^1–3^, we investigated the “seed” function of Ahnak. Therefore, we established B16F10 cells with stable knockdown of Ahnak (B16F10-shAhnak). N-cadherin expression, migration, and invasion were attenuated in B16F10-shAhnak cells compared to B16F10-shControl cells. Moreover, lung metastasis was significantly suppressed after the injection of B16F10-shAhnak #7 cells into C57BL/6 mice (Fig. 5A,B). The key early event associated with metastasis is EMT which results from a loss of E-cadherin, disruption of cell-cell junction and acquisition of invasive pseudopodial structure^1–3^. Shankar *et al*. reported identification of Ahnak as a pseudopod-specific protein in six metastatic human tumor cell lines (metastastic prostate cancer cell line Du145, breast cancer cell line MDA-231 and MDA-435, fibrosarcoma HT1080, and glioma U251 and U87 cells)^18^. Keshamounl *et al*. reported results from iTRAQ-2DLC-MS/MS analysis which indicated induction of Ahnak expression during TGFβ-induced EMT in human lung cancer cells (A549)^20^. Up-regulation of Ahnak is associated with increased invasion and metastasis of human lung cancer cells. Moreover, Ahnak has been identified as a marker for sarcomatoid mesothelioma^17,25^. Two reports showed that Ahnak expression is correlated with cell migration and invasive ability in malignant mesothelioma cells. Dumitru *et al*. showed that Ahnak expression in tumor tissues from 83 larynx carcinoma patients was up-regulated and tumoral Ahnak overexpression is significantly associated with poor survival of these patients^24^. In sum, multiple previous reports and our results together strongly indicate that Ahnak plays an important role in EMT process during intravasation of tumor metastasis.

Here, we report the signaling mechanism that illustrates the significance of Ahnak in malignant tumor (Fig. 5C). Our results shed light on molecular mechanisms of metastasis of various cancer tissues including lung cancer, head and neck cancer, and mesothelioma. Most importantly, Ahnak promotes TGFβ-induced EMT and cancer metastasis. This in turn implies that analyses of dysregulated Ahnak expression would be essential for understanding cancer development and progression. Our study thus points to the value of Ahnak as a prognostic marker for malignant tumors and an important therapeutic target for cancer treatment.

## Methods

### Materials

Recombinant human TGFβ1 was purchased from R&D Systems. An antibody against Ahnak was generated by using a synthetic KIS peptide according to a previous report by Young In Frontier (Rep. of Korea). The following antibodies were also used: N-cadherin (Abcam), β-catenin (Santa Cruz Biotechnology), Smad3 (Zymed Laboratories), and phosphorylated Smad3 (Cell Signaling). Tetramethyl rhodamine isothiocyanate (TRITC)-labelled mouse and rabbit secondary antibodies were purchased from Kirkegaard & Perry Laboratories (KPL). All experimental protocols were approved by the ethical guidelines of Institutional Animal Care and Use Committee (IACUC) of Ewha Womans University (EWU) and carried out in accordance with local guidelines and regulations.

### Cell culture and siRNA transfection

The HaCaT human keratinocyte and B16F10 mouse melanoma cell lines were grown in Dulbecco’s modified Eagle’s medium (DMEM) supplemented with 10% fetal bovine serum (FBS, WELGENE, Rep. of Korea) and 1% (v/v) antibiotic-antimycotic solutions (GIBCO). Human aortic endothelial cells (HAECs) were purchased from Lonza and grown to confluence in endothelial growth medium (EGM2) supplemented with 2%FBS, 0.1% GA-1000, ascorbic acid, heparin, VEGF, hEGF, hFGF-B, R3-IGF-1 and hydrocortisone. For lentivirus generation, the lentiviral vectors pGIPZ-shControl and pGIPZ-shAhnak were transfected into 293 T cells. Following a 24-h incubation, the medium containing virus particles was harvested after an additional 24 h, 48 h and 72 h. The supernatant was collected, passed through a 0.45μm filter, and concentrated by ultracentrifugation at 35,000 rpm for 4 h. The pellets were resuspended in serum-free medium. B16F10 melanoma cells stably infected with the shAhnak lentivirus were established by cultivating B16F10 cells in the presence of puromycin for 25 days, with media change every 3–4 days. Subsequently, colonies were picked and used to generate monoclonal cell lines. Control siRNA (siControl, ON-TARGETplus Non-targeting Control siRNA D-001810-02) and human Ahnak siRNA (siAhnak, ON-TARGETplus siRNA L-014285-01) were purchased from Dharmacon. Transfection was performed using 50 nM of siRNA and Lipofector-EZ (AptaBio, Korea) according to the manufacturer’s protocol.

### RNA isolation and quantitative real-time PCR (qRT-PCR)

Total RNA was isolated with Trizol reagent. cDNA was synthesized using 1 μg of RNA per 20 μL reaction volume with the Reverse Transcription System (Promega) according to the manufacturer’s protocol. qRT-PCR was performed in duplicate with 2 μL of cDNA per well. TaqMan® Gene Expression Assays (Applied Biosystems) and KAPA PROBE FAST Universal 2X qPCR Master Mix (KAPA BIOSYSTEMS) were employed to analyze Ahnak expression. The expression of other genes was quantified with KAPA SYBR® FAST Universal 2X qPCR Master Mix (KAPA BIOSYSTEMS) using the following primers:Snail, 5′-GGCAGCTATTTCAGCCTCCT-3′ and 5′-CATCGGTCAGACCAGAGCAC-3′;Slug, 5′-TCAAGAAGCATTTCAACGCC-3′ and 5′-CTGTGGTCCTTGGAGGAGGT-3′;Twist1, 5′-GACAGTGATTCCCAGACGGG-3′ and 5′-GCTGATTGGCACGACCTCTT-3′;CTGF, 5′-TTAGCGTGCTCACTGACCTG-3′ and 5′-GCCACAAGCTGTCCAGTCTA-3′;COL1A2, 5′-CGGAGGTATGCAGACAACGA-3′ and 5′-CACGGGGCTGGCTTCTTAAA-3′; TIMP1, 5′-CTTCTGCACTGATGGTGGGT-3′ and 5′-TAAATGTCCACGCTAGGGGC-3′; ICAM1, 5′-AGCCCAAGTTGTTGGGCATA-3′ and 5′-AGTCCAGTACACGGTGAGGA-3′; VCAM1, 5′-CATGGAATTCGAACCCAAACA-3′ and 5′-TTTCGGAGCAGGAAAGCCCTGG-3′; and GAPDH, 5′-CTCCTGTTCGACAGTCAGCC-3′ and 5′-ACCAAATCCGTTGACTCCGAC-3′

The relative expression of each gene was analyzed by the 2−ΔΔCt method. The mean ± standard deviation (S.D.) and statistical significance (Student’s t-test) were calculated based on three independent experiments.

### Monolayer wound healing assay

HaCaT cells (1 × 10^5^) were plated on a 6-well culture dish and transfected with the indicated siRNA. Ahnak-depleted B16F10 stable cells (2 × 10^5^) were plated on a 6-well culture dish. After serum starvation (12~16 h), cells were treated with 10 μg/mL mitomycin C (Sigma) for 3 h at 37 °C to block proliferation. Cells were washed with PBS and wounded by scratching with a 200 μL pipette tip. Cells were washed again to remove detached cells. The wounded cells were incubated in serum-free DMEM in the presence or absence of 5 ng/mL TGFβ for 24 h. At 0 h and 24 h, three different regions per well were photographed using an Axiovert 40 C inverted microscope (Carl Zeiss). The number of migrated cells was counted in three regions per well, and the mean ± S.D. of three independent experiments was calculated to graphs. Statistical significance was analyzed with Student’s t-test. To confirm Ahnak expression, cells were lysed and western blot analysis was performed as described above.

### Transwell migration and matrigel invasion assays

Prior to these assays, Matrigel^TM^ Basement Membrane Matrix was purchased from BD Biosciences and diluted to 1 mg/mL with cold serum-free DMEM. The Ahnak siRNA-transfected HaCaT cells and Ahnak-depleted B16F10 stable cell lines were serum deprived and treated with 10 μg/mL of mitomycin C in serum-free DMEM was treated for 3 h. Cells (2 × 10^4^) were seeded in the presence or absence of TGFβ (5 ng/mL) onto duplicate matrigel (100 μg)-coated Transwell® Permeable Supports (Costar, 24 well plate with 8 μm pore size). Lower chambers were filled with DMEM containing 10% FBS as the chemoattractant. Cells were allowed to invade for 48 h at 37 °C. The remaining cells were lysed with RIPA buffer and subjected to western blot analysis. After removing the cell on the upper side of membrane, the invading cells were stained with 0.05% crystal violet or hematoxylin and eosin after fixation in 3.5% p-formaldehyde. Stained cells were photographed using an Axiovert 40 C inverted microscope (Carl Zeiss). The number of invasive cells per frame was counted in every duplicated well. After three independent experiments, invasive cell number was calculated and graphed as the mean ± S.D. Statistical significance was calculated using Student’s t-test.

### Lung metastasis study

Ahnak-knockdown B16F10 stable cells and control B16F10 cells were prepared to inject 5 × 10^5^ cells per mouse in 100 μL of PBS. For tail vein injections, 6-to-8-wk-old male mice were restrained utilizing a rodent restrainer. The mouse tail was kept in contact with a 40 °C heat pad to relax the vein just before injection. Cells were injected into the mouse using a 30.5-gauge needle. Fourteen days later, mice were sacrificed and perfused with PBS through the right ventricle until pulmonary blood was completely removed. Lung tissues were preserved in 10% neutral buffered formalin (NBF), and tumor colonies on the lung surface were counted. GraphPad Prism software was used for statistical assessments and graphical presentations of colony number. Median values and statistical significance were obtained with Student’s t-test and the Mann-Whitney U test. All animal procedures were approved by Institutional Animal Care and Use Committee (IACUC) of Ewha Womans University (EWU) and performed in accordance with the ethical guidelines and regulation of IACUC of EWU.

### Statistics

Statistical analysis was performed with a two-tailed unpaired t-test. Data are presented as the mean ± SD of values obtained from three to five independent experiments. All western blots in figures are representative of three independent experiments.

## Electronic supplementary material

Supplementary Information
