# Supplementary material for: Ahnak promotes tumor metastasis through transforming growth factor-β-mediated epithelial-mesenchymal transition
Source: Sci Rep. 2018 Sep 26;8:14379. doi: 10.1038/s41598-018-32796-2 (PMC6158194; doi:10.1038/s41598-018-32796-2)
Supplement: Supplementary file 1 — Supplementary Information [file 41598_2018_32796_MOESM1_ESM.pdf]

**Supplementary Information**

# Ahnak promotes tumor metastasis through transforming growth factor-β-mediated epithelial-mesenchymal transition

Mira Sohn[*], Sunmee Shin[*], Jung-Yeon Yoo, Yookyung Goh, In Hye Lee [#], and Yun Soo Bae[#]

Department of Life Sciences, Ewha Womans University, Seoul 120-750, Korea

[*]These authors contributed equally to this work.

[#] Corresponding Author: Yun Soo Bae, Department of Life Science, Ewha Womans University, Seoul 120-750, Korea. baeys@ewha.ac.kr, phone: 82-2-3277-2729, fax: 82-2-3277-3760; In Hye Lee, Department of Life Science, Ewha Womans University, Seoul 120-750, Korea. lih3026@ewha.ac.kr, phone: 82-2-3277-3032, fax: 82-2-3277-3760

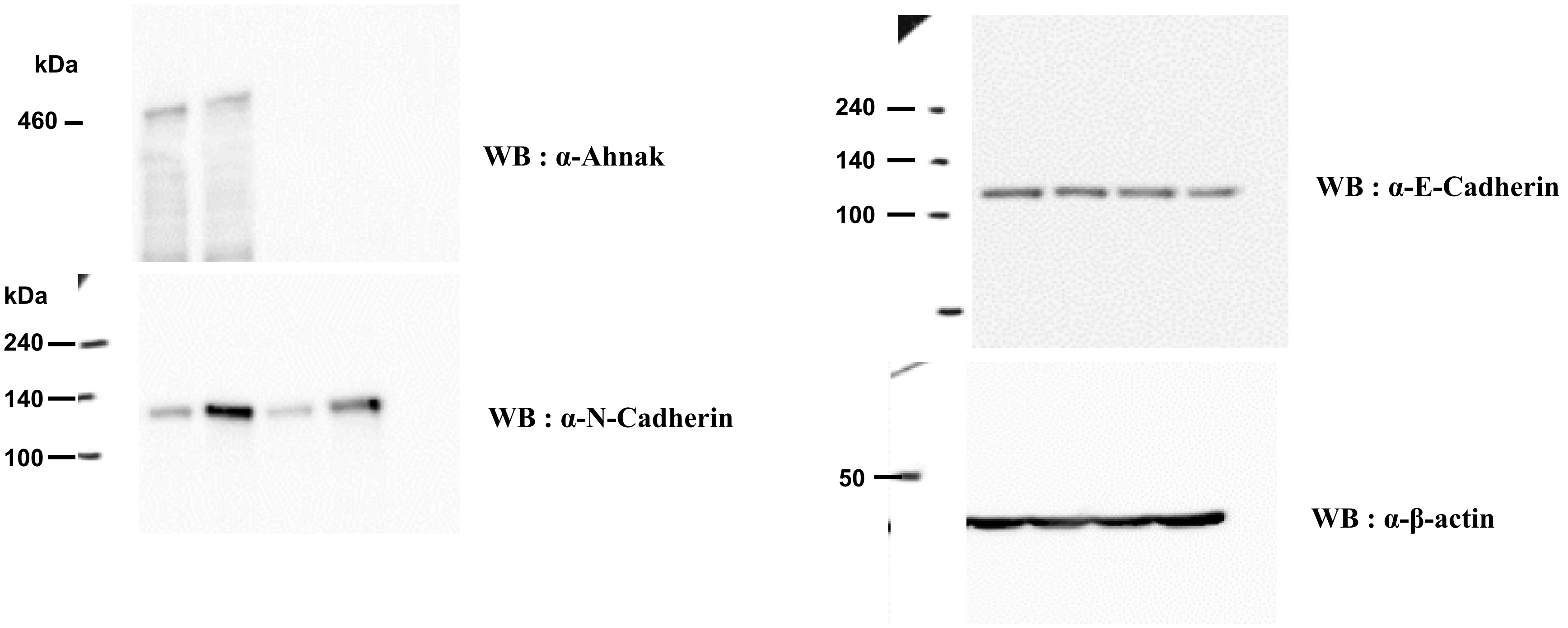


**Supplementary Figure S1. Requirement of Ahnak for TGFβ-induced EMT**

HaCaT cells were transfected, serum deprived, and stimulated with TGFβ (5 ng/mL) for 72 h. Cells were lysed and subjected to western blot (WB) analysis with the indicated antibodies.

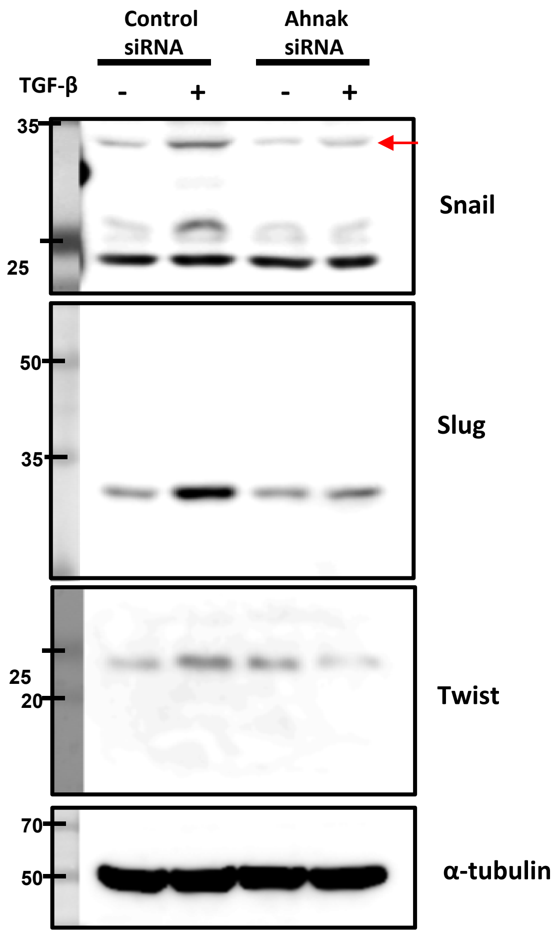


**Supplementary Figure S2. TGFβ-induced EMT markers expression in knockdown expression of Ahnak in HaCaT cells**
HaCaT cells were transfected, serum deprived, and stimulated with TGFβ (5 ng/mL) for 24 h. Cells were lysed and subjected to western blot (WB) analysis with the indicated antibodies.

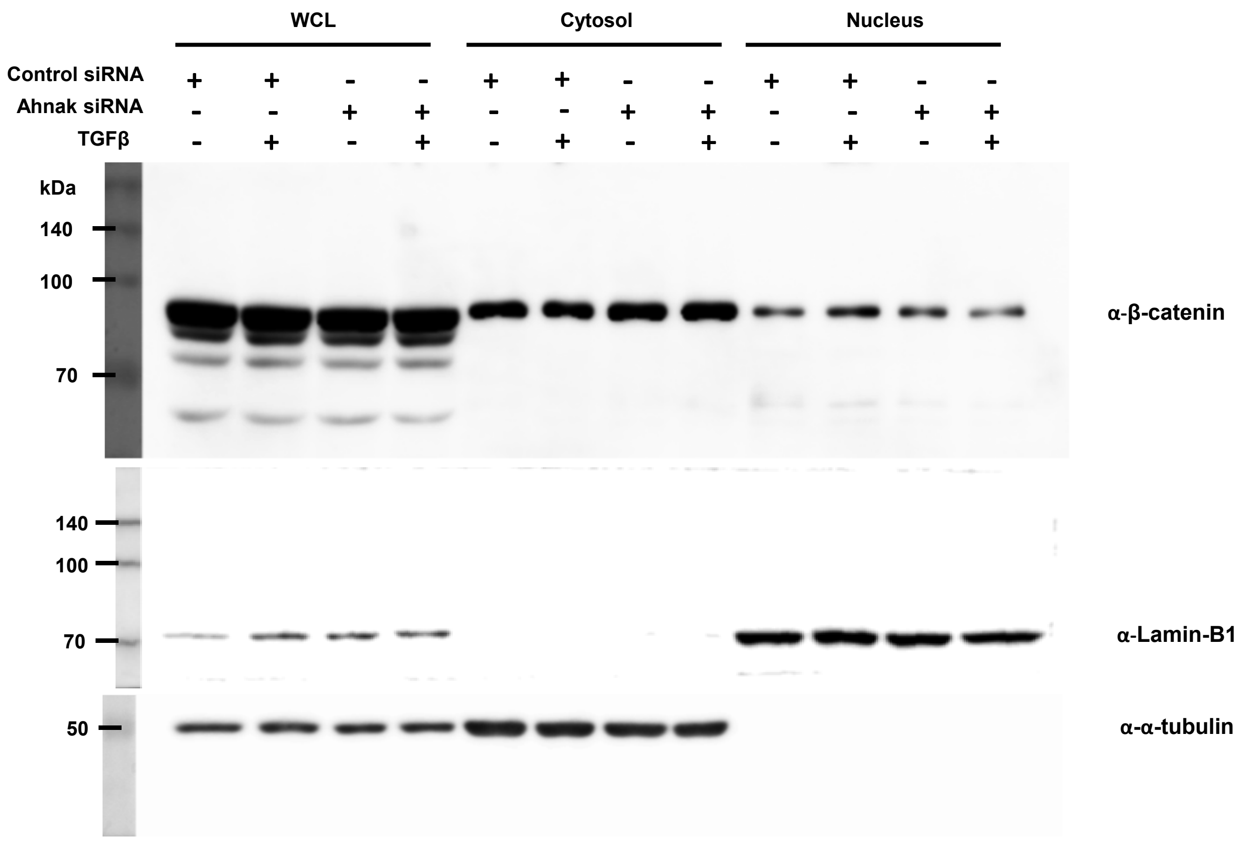


**Supplementary Figure S3. Effect of Ahnak on TGFβ-induced β-catenin nuclear localization in HaCaT cells**

HaCaT cells ($6x10^4$) cultured in 100mm culture dishes were transfected control siRNA or Ahnak siRNA as described above. HaCaT cells were serum starved for 16h and then incubated with or without TGF-β (5ng/ml) for 1h. The nuclear fraction was isolated by the cell fractionation kit (Cell signaling; #9038) according to manufacturer's instruction.

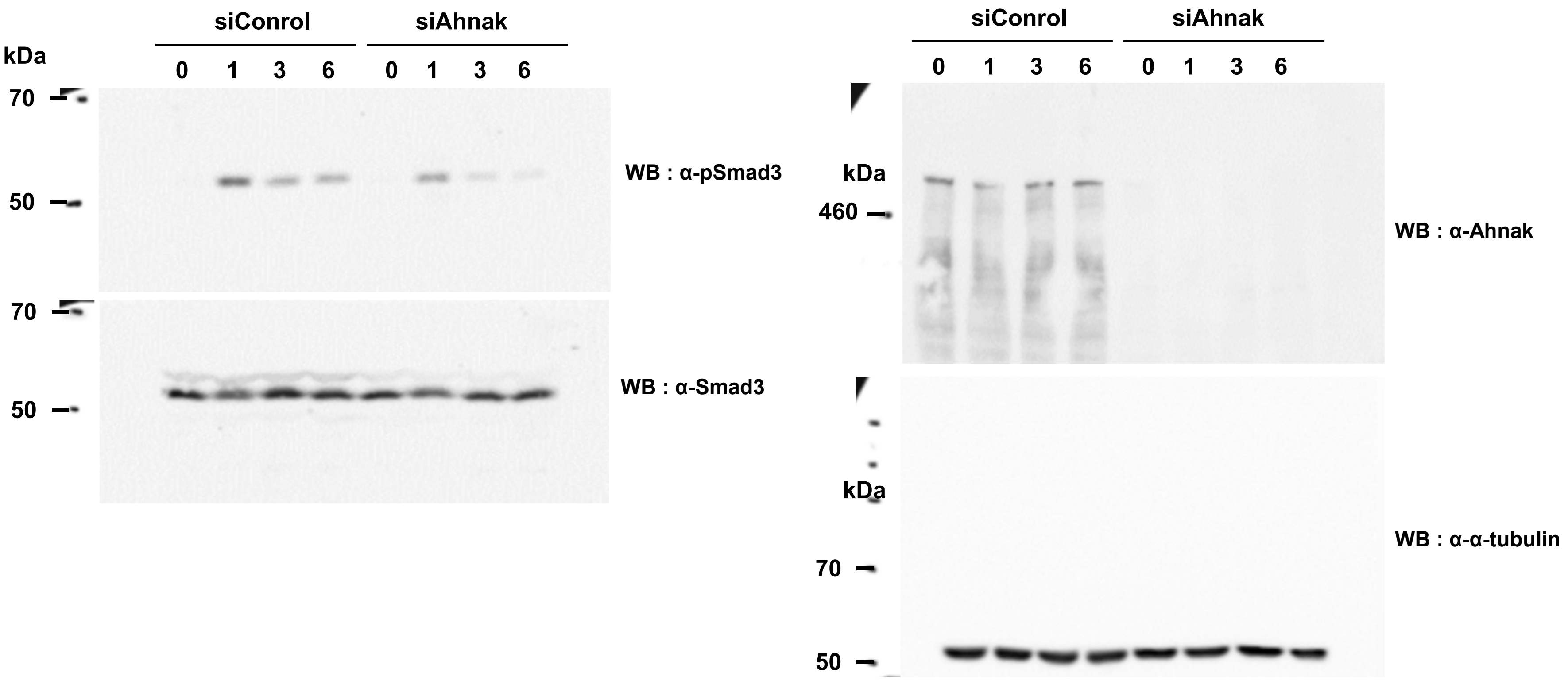


**Supplementary Figure S4. Activation of TGFβ/Smad3 signaling by Ahnak**

HaCaT cells were transfected with the indicated siRNA, serum starved, and stimulated with TGFβ (5 ng/mL) for the indicated time. Cells were lysed and subjected to western blot (WB) analysis with antibodies to p-Smad3, Smad3, Ahnak and actin.

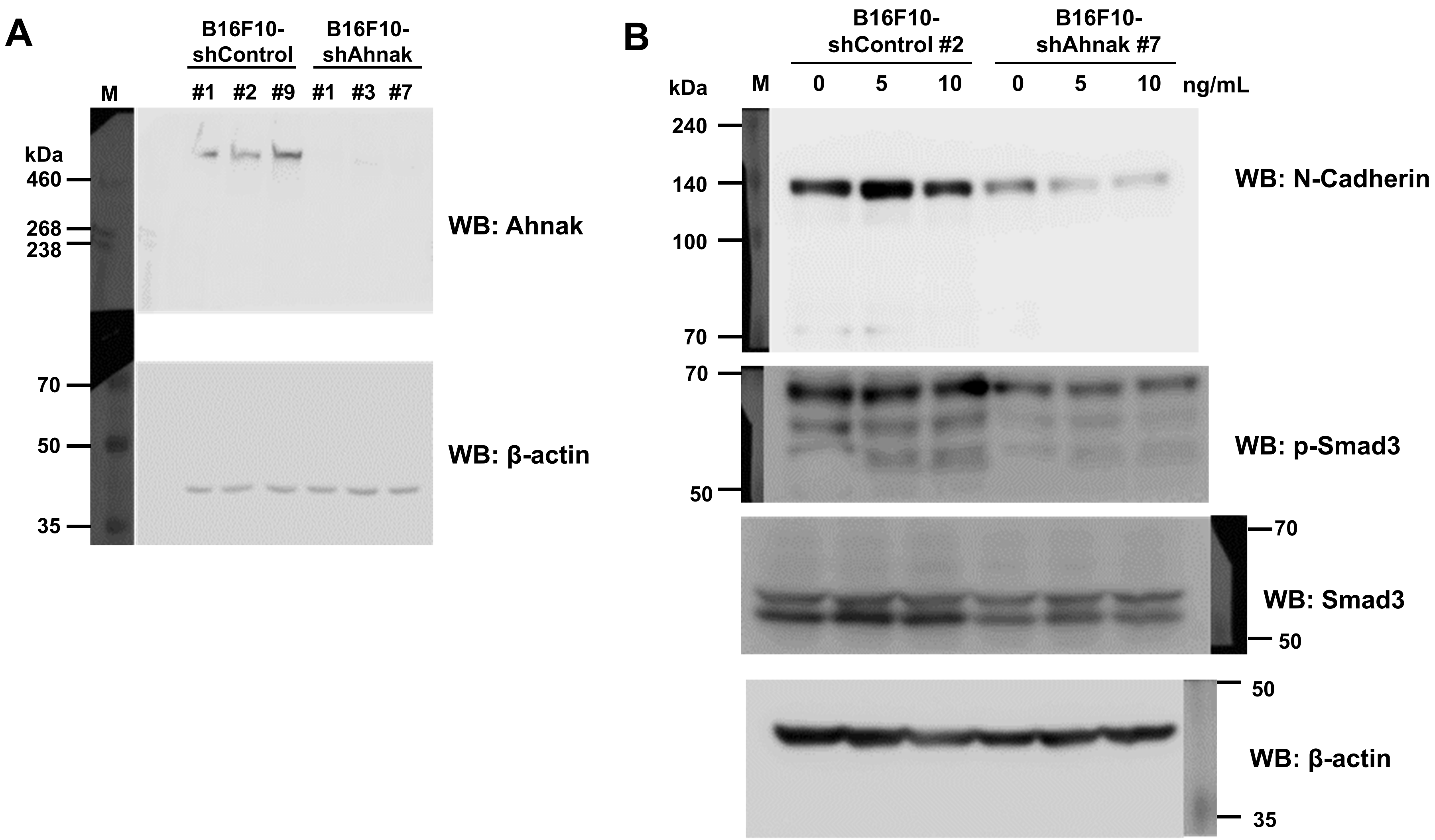


**Supplementary Figure S5. Ahnak regulates EMT-mediated melanoma cell migration and invasion**

(A) B16F10 melanoma cells were stably infected with pGIPZ lentivirus-shControl and shAhnak and selected by colony picking to generate monoclonal cell line. Each cell line was subjected to western blot (WB) analysis with the indicated antibodies**. (B)** Ahnak-depleted B16F10 stable cells were serum starved and stimulated with TGFβ (5 ng/mL) for 24 h. Cells were lysed and subjected to western blot (WB) analysis with the indicated antibodies.

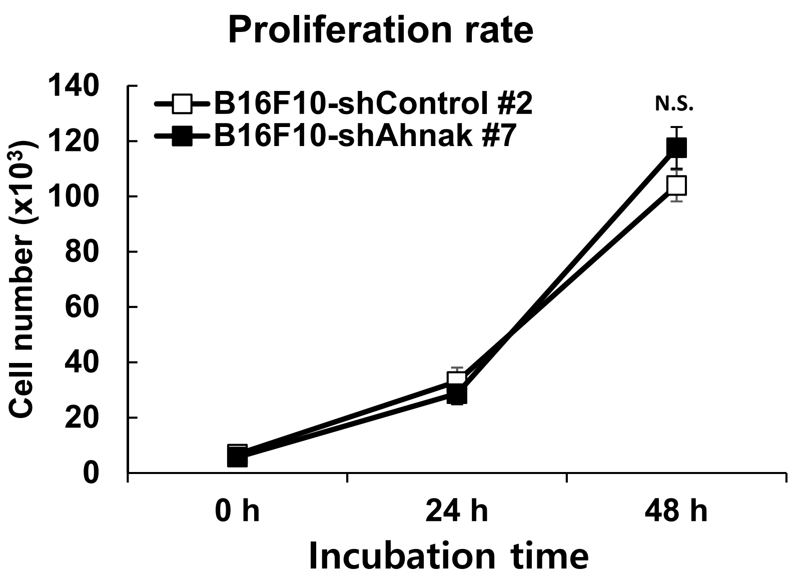


**Supplementary Figure S6. Proliferation rate of B16F10-shControl #2 and B16F10-shAhnak #7 cells**

Both cell lines (B16F10-shControl #2 and B16F10-shAhnak #7 cells) were cultured in 96well plates for the indicated incubation time. The cells were harvested and counted. The data are means ± SEM of three independent experiments.
